# The knowledge, attitudes, and practices of smallholder cattle farmers concerning the epidemiology of bovine fasciolosis in the North West Province, South Africa

**DOI:** 10.1007/s11250-023-03478-7

**Published:** 2023-02-25

**Authors:** Sunday Charles Olaogun, Geoffrey Theodore Fosgate, Charles Byaruhanga, Munyaradzi Christopher Marufu

**Affiliations:** 1 Department of Production Animal Studies, University of Pretoria, Private Bag X4, Onderstepoort, Pretoria , 0110 South Africa; 2 Department of Veterinary Medicine, Faculty of Veterinary Medicine, University of Ibadan, Ibadan, 200005 Nigeria; 3 Department of Veterinary Tropical Diseases, University of Pretoria, Private Bag X4, Onderstepoort, Pretoria, 0110 South Africa

**Keywords:** Beef production, Epidemiology, Liver fluke, Perceptions

## Abstract

Bovine fasciolosis has negative impacts on cattle production worldwide, more so on the African continent and especially in smallholder farming areas with limited level of awareness. A cross-sectional questionnaire-based survey was conducted to investigate the knowledge, attitudes, and practices concerning bovine fasciolosis among smallholder cattle farmers in the North West Province of South Africa. A total of 153 farmers were interviewed from three villages of the Moretele Local Municipality in Bojanala District. The majority of respondents were male (84%) farm owners (81%) that had low education levels (56% primary school or less) and employed extensive cattle management systems (84%). A large number of farms lacked infrastructure including calving pens (88%), restraining equipment (85%), and weight determination equipment (92%) while sourcing drinking water for cattle from rivers or dams (58%). No evaluated factors were significantly associated with a positive fasciolosis epidemiological knowledge score. However, education level (*P* = 0.046), some cattle breeds (*P* = 0.022), and management system (*P* < 0.001) of the smallholder farmers were associated with a positive practice score concerning bovine fasciolosis prevention. We therefore recommend that education programs be introduced that focus on the mode of transmission, risk factors, zoonotic importance, and practices associated with the prevention and control of bovine fasciolosis.

## Introduction

Many rural communities in Africa make their livelihoods from cattle production, which also provides the essential dietary components of milk and meat (Kabubo-Mariara 2009). The livestock sector contributes more than 40% to the gross domestic product (GDP) of South Africa’s agricultural economy (Masemola et al. 2019). Smallholder cattle farmers, defined as poorly-resourced farmers with small plots for the rearing of cattle both for household food and for nutritional security (Udo et al. 2011), depend on this sector for their livelihoods (Rootman et al. 2015). The roles of cattle for smallholder farmers include sociocultural (traditional ceremonies, sacrifice purposes), economic (family financial base, property protection, livelihood), and sustainable agricultural production purposes (traction for tillage, manure as fertilizer for crops, agricultural diversification) (Ndoro et al. 2014). Notwithstanding these benefits, smallholder cattle production is constrained by a number of factors, chief among which are parasitic diseases. Fasciolosis (liver fluke infection) is considered the most important parasitic disease and a major impediment to sustainable cattle production (Bayer et al. 2003).

Liver fluke infection is a neglected tropical disease (parasitic zoonosis) of animals (fasciolosis) and people (fascioliasis). Infestation with *Fasciola hepatica* and/or *Fasciola gigantica* liver flukes is the cause of disease, and intermediate snail hosts are required for the pre-parasitic developmental phase of these parasites (Lalor et al. 2021). Factors including climatic conditions (adequate moisture and temperature) and the presence of definitive mammalian hosts are also essential for the completion of the parasite’s life cycle (Fairweather 2011). The importance of host attributes (sex, age and breed) and seasonal risk factors for fascioliasis in domestic ruminants has been previously described (Islam et al. 2014). The roles played by vegetation and water plant species in the transmission of fasciolosis, especially human fascioliasis, have long been established in most developed countries (Mas-Coma et al. 2018). In cattle, fasciolosis causes anemia and hypoproteinemia, which contribute to herd morbidity and mortality. Additional effects on cattle production include reduced milk yield, poor growth and reproductive performance, and increased production costs due to required treatments (Beesley et al. 2018). The treatment for clinical fascioliasis is anthelmintic therapy, specifically triclabendazole, a member of the benzimidazole group (Merachew and Alemneh 2020).

Fasciolosis has been reported to have higher prevalence in cattle herds reared by smallholder farmers due to high illiteracy rates, poor recognition of the disease, limited resources for control, suboptimal nutrition, and poor biosecurity (Nyindo and Lukambagire 2015). Poor off-take and reduced incomes are characteristic of smallholder cattle operations (Molefi and Mbajiorgu 2017). It is essential to control bovine fasciolosis in smallholder cattle herds, and attention should be given to the farmers’ perceptions and practices concerning the disease, as these will affect the success of implemented control measures.

Studies on knowledge, attitudes, and practices (KAP) of smallholder farmers on bovine fasciolosis have been used to assess their willingness to adopt prevention and control measures (Tiongco et al. 2012). Inadequate knowledge of the disease, the presence of multiple high-risk farm practices, and inappropriate perceptions and bad practices require education for improvement. Assessment of farmers’ KAP on bovine fasciolosis is essential for the development of appropriate policies and strategies to prevent and control the disease (Aregahagn and Melkamu 2018). The current study was, therefore, aimed at assessing smallholder cattle farmers’ knowledge and awareness of risk factors, zoonotic importance, transmission, prevention, and control of bovine fasciolosis in the North West Province of South Africa.

## Materials and methods

### Description of the study site

**Fig. 1:**
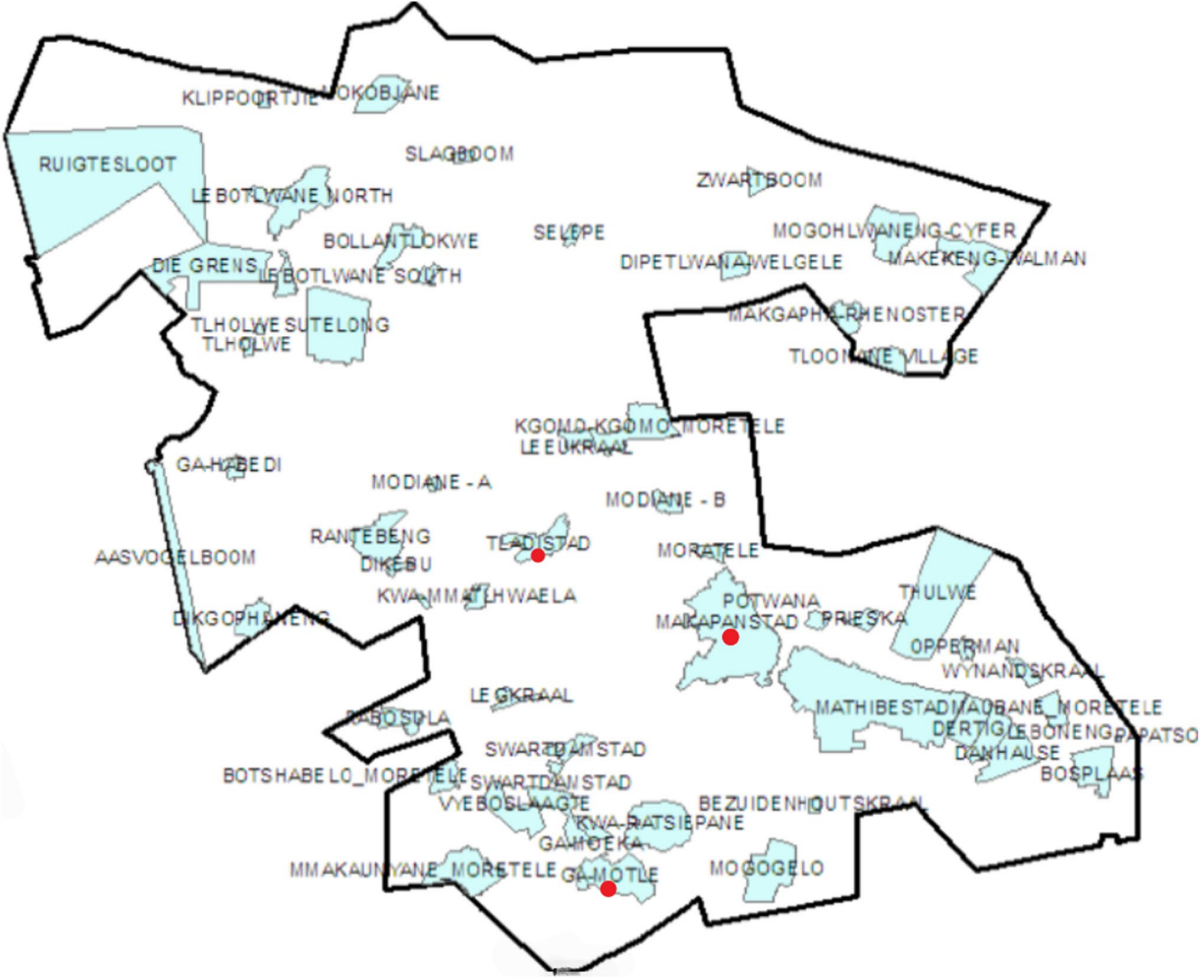
Map of Moratele Local Municipality showing the three study sites marked in red. Adapted from Maime (2015)

The study was conducted in three villages (Makapanstad, Ga-Motle, and Tladistad) in the Moretele Local Municipality, falling under the Bojanala District Municipality in the North West Province of South Africa (Fig. 1). Makapanstad is located at 25° 14′ 36″ South and 28° 7′ 19″ East and has a total area of 20.45 km^2^ and a human population of 15,000. Ga-Motle is located at 25° 21′ 14″ South and 28° 4′ 9″ East and encompasses an area of 8.3 km^2^ with a human population of 5600. Tladistad is located at 25° 12′ 10.8″ South and 28° 2′ 6″ East, with an area of 3.30 km^2^ and a human population of 3000 (Letsoalo et al. 2000).

### Sample size determination

This was based on the formula given by Thrusfield (2007) using a simple random sampling technique, with 5% absolute precision, and estimated prevalence was set as 11% (based on previous experience of one of the authors that has done surveys in the area 6 months prior to the study).

$$n = {1.96}^{2}(\mathrm{Pexp}) (1-\mathrm{Pexp})/\mathrm{ d}2$$

where *n* = total number of sample size; *d* = absolute precision; *Pexp* = expected prevalence; *n* = unknown; *d* = 5% = 0.05; and *Pexp* = 11%. A sample size of approximately 153 smallholder cattle farmers represented by animal owners and handlers in various farms was obtained.

### Farmer selection and data collection

The district and local municipality were selected based on the willingness of farmers to participate in the study, the availability of cattle, and the presence of semi-intensive and extensive smallholder cattle farmers. The three studied villages were selected based on farmers’ location accessibility and geographical spread in addition to the above stated criteria for district and local municipality selection. Smallholder farmers were selected using a snowball sampling technique (Qokweni et al. 2020). Inclusion criteria were active smallholder cattle farmers owning more than four animals, which consented to participation, and were at least 18 years old.

A paper-based questionnaire was pre-tested and then administered to a total of 153 farmers in the villages of Makapanstad (*n* = 62), Ga-Motle (*n* = 41), and Tladistad (*n* = 50). Informed consent was obtained before the interviews, and respondents were assured that their identity and responses would not be disclosed. The questionnaire sought information regarding farmers’ and cattle herd demographics; farm infrastructure; and farmers’ knowledge, attitudes, and practices concerning the epidemiology of bovine fasciolosis. Information including sex, age, language, years of rearing, and marital status were requested on the questionnaire. The questionnaire involved four major sections (A, B, C, and D) with subsections containing 39 major questions and several questions under each major question. The estimated time of completion of the questionnaire was 30 min. Section A requested information on farmers’ demographic information, and section B requested herd structure demography information. Section C requested information on farmers’ level of knowledge/awareness on clinical signs, mode of transmission, zoonotic importance, and risk factors of bovine fasciolosis. Section D requested information on practices associated with the prevention and control of bovine fasciolosis.

### Statistical analyses

Data were entered into a Microsoft Excel® (Microsoft Corporation, USA) spread sheet and then analyzed using the Statistical Package for Social Scientists (SPSS Version 26). Descriptive statistics were used to present data on farmer and herd demography, farm characteristics, and management practices. The association between location and farmer and herd demographic, farm structure, and management variables was determined using chi-square tests. The epidemiology knowledge and practice scores were established through scoring of the responses based on coding of the questionnaire. Correct responses were scored as + 1, and incorrect responses were coded as − 1 and unsure coded as 0. These were inputted on the spread sheet and formulas entered to sum up the total scores concerning epidemiological knowledge (questionnaire section C) and beneficial fasciolosis practices (questionnaire section D). Total scores greater than 0 were considered indicative of positive epidemiological knowledge and fasciolosis practices, respectively. Binary logistic regression was used to investigate the association of potential predictors and having positive knowledge and practices independently. Univariate screening models were fit, and all predictors with Wald *P* < 0.2 were selected for multivariable modeling. Multivariable models were fit using a manual backwards elimination process starting with all variables identified in the univariate screening models. Variables were removed one-by-one based on the largest Wald *P* value until all remaining variables were *P* < 0.05. The fit of the final model was assessed using a Hosmer–Lemeshow test. Odd ratios (OR) and *P* values were used to estimate the level of association and statistical significance, respectively. OR were calculated with 95% confidence intervals (CI), and *P* < 0.05 was used to determine statistical significance.

## Results

### Farmer and herd demographic information and farm infrastructure

The majority of farmers were males (129/153) and most of the respondents owned their farm (124/153). A larger proportion of the farmers had no formal or completed only primary education (86/153). Most of the farmers were married (120/153), and the majority had more than 10 years’ cattle-rearing experience (125/153). A large proportion of farmers practiced extensive cattle management (128/153) (Table 1).

**Table 1:** The association between location and potential categorical predictors of 153 smallholder cattle farmers in communal areas of North West Province South Africa from June to Oct 2019

Variables	Total (*n* = 153)	Village M (*n* = 62) Frequency	% (95%CI)	Village T (*n* = 41) Frequency	% (95%CI)	Village G (*n* = 50) Frequency	% (95%CI)	*P* < value^*^
Farm ownership								
Owner	124	47	76 (64–85)	33	80 (66–90)	44	88 (76–94)	0.261
Hired hand	29	15	24 (15–36)	8	20 (10–34)	6	12 (7–24)	
Sex								
Male	129	51	82 (71–90)	34	83 (69–91)	44	88 (76–94)	0.680
Female	24	11	18 (10–29)	7	17 (9–31)	6	12 (7–24)	
Marital status								
Single	15	9	15 (8–25)	3	7 (3–19)	3	6 (2–16)	0.139
Married	120	50	81 (69–89)	32	78 (63–88)	38	76 (63–86)	
Divorced/widow	18	3	5 (2–13)	6	15 (9–28)	9	18 (10–31)	
Education level								
No formal education	23	9	15 (8–25)	4	10 (4–23)	10	20 (11–33)	0.388
Primary	63	22	35 (25–48)	18	44 (30–59)	23	46 (33–60)	
Secondary/tertiary	67	31	50 (38–62)	19	46 (32–61)	17	34 (22–48)	
Language								
Sepedi	59	29	47 (35–59)	10	24 (14–39)	20	40 (28–54)	0.147
Xhosa	12	5	8 (3–18)	5	12 (5–26)	2	4 (1–13)	
Tswana	82	28	45 (33–57)	26	63 (48–76)	28	56 (42–69)	
Management system								
Backyard	44	31	50 (38–62)	4	10 (4–23)	9	18 (10–30)	< 0.001
Extensive	84	28	45 (33–57)	26	63 (48–76)	30	60 (46–72)	
Semi-intensive	25	3	5 (2–13)	11	27 (16–42)	11	22 (13–35)	
Farming experience								
Less than 10 years	28	16	26 (17–38)	7	17 (9–31)	5	10 (4–21)	0.158
10 to 20 years	76	29	47 (35–59)	23	56 (41–70)	24	48 (35–61)	
More than 20 years	49	17	27 (18–40)	11	27 (16–42)	21	42 (29–56)	

Multiple and other cattle breeds (94/153) were most common followed by Brahman (42/153) and lastly Nguni, Bonsmara, or non-descript (17/153). Farmers reported the body condition score of their cattle to be mostly average (69/153), followed by poor (63/153) and lastly good (21/153). Most farmers solely grazed their cattle on pastures (109/153), while fewer included feed supplements with pasture grazing (44/153). Many farms lacked infrastructure including calving pens (134/153), restraining equipment (130/153), and weight determination equipment (140/153). Most farmers (89/153) sourced drinking water from rivers or dams followed by wells (24/153) and municipality water (16/153). Sixteen percent (24/153) of farmers used more than one water source (Table 2).

**Table 2:** The association between locations and potential categorical predictors in the herd structure of smallholder cattle farmers in communal areas of North West Province South Africa from June to Oct 2019

Variables	Total	Village M (*n* = 62) Frequency	% (95%CI)	Village T (*n* = 41) Frequency	% (95%CI)	Village G (*n* = 50) Frequency	% (95%CI)	*P* value^*^
Herd structure								
Single	150	61	98 (91–100)	40	98 (87–100)	49	98 (90–100)	0.957
Multiple	3	1	2 (0.2–9)	1	2 (0.4–13)	1	2 (0.4–10)	
Cattle breed								
Brahman	42	15	24 (15–36)	15	37 (24–52)	12	24 (14–37)	0.133
Nguni, Bonsmara, or non-descript	17	9	15 (8–25)	6	15 (7–28)	2	4 (1–13)	
Multiple breed or other	94	38	61 (49–72)	20	49 (34–64)	36	72 (58–83)	
Body condition score								
Poor	63	18	29 (19–41)	21	51 (36–66)	24	48 (35–61)	0.099
Average	69	34	55 (43–67)	17	41 (28–57)	18	36 (24–50)	
Good	21	10	16 (9–27)	3	7 (3–19)	8	16 (8–29)	
Type of feed								
Pasture	104	44	71 (59–81)	29	71 (56–82)	31	62 (48–74)	0.544
Mixed feed	49	18	29 (19–41)	12	29 (18–44)	19	38 (26–52)	
Drinking water source								
Dam/river	89	30	48 (36–61)	24	59 (43–72)	33	66 (52–78)	< 0.001
Municipal water	16	9	15 (8–25)	6	15 (7–28)	3	6 (2–16)	
Well	24	20	32 (22–45)	2	5 (1–16)	2	4 (1–13)	
Multiple	24	3	5 (2–13)	9	22 (12–37)	12	24 (14–37)	
Calving pen								
Yes	19	8	13 (7–23)	3	7 (3–19)	8	16 (8–29)	0.453
No	134	54	87 (77–93)	38	93 (81–97)	42	84 (71–92)	
Restraining equipment								
Yes	23	8	13 (7–23)	7	17 (9–31)	8	16 (8–29)	0.823
No	130	54	87 (77–93)	34	83 (69–91)	42	84 (71–92)	
Weighing equipment								
Yes	13	5	8 (3–18)	1	2 (0.4–13)	7	14 (7–26)	0.142
No	140	57	92 (82–97)	40	98 (87–100)	43	86 (74–93)	

Farmer cattle management system varied by study village (*P* < 0.001), but none of the other evaluated demographic variables was significant (*P* > 0.05; Table 1). Ga-Motle (11/41) and Tladistad (11/51) had higher proportions of semi-intensive farmers compared to Makapanstad (3/62). The source of drinking water varied by study village (*P* < 0.001), but none of the other evaluated categorical predictors differed by location (*P* > 0.05; Table 2). Dam or river water was the most common source across all locations followed by well water in Makapanstad, as opposed to multiple water sources in Ga-Motle and Tladistad.

### Predictors of positive epidemiological knowledge and practices

There were no significant associations between farmers’ location, ownership status, sex, age, educational level, years of experience, languages, cattle breeds being reared, and system of management and their feed sources with positive epidemiological knowledge and practice scores of fasciolosis. This implies that none of these variables is a predictor of the smallholder farmers’ positive epidemiological knowledge of fasciolosis (Table 3). Farmers’ ownership status, age, marital status, level of education, years of rearing experience, cattle breed being reared, animals’ water sources, and farmers equipment status did not have any significant association with positive practice scores about bovine fasciolosis. However, there were significant associations (*P* < 0.05) between positive practice scores and some categorical predictors. Farmers in village T had higher likelihood of a positive practice score compared to village G, while male farmers and farmers practicing extensive system of management had significantly lower likelihood of positive practice scores compared to female farmers and farmers with a semi-intensive system of management, respectively. Farmers practicing backyard system also possessed a significantly lower positive practice score compared to the semi-intensive farmers. Farmers practicing grazing only also had a significantly lower positive practice compared to those practicing mixed or concentrate feeding (Table 4). Multivariable modeling identified education level (*P* = 0.046), cattle breed (*P* = 0.022), and farmers’ system of management (*P* = 0.001) as independent predictors of positive practice scores concerning bovine fasciolosis prevention. All other variables such as farmers’ gender, age, marital status, years of rearing experience, cattle breed being reared, animals’ water sources, and farmers’ equipment status cannot be taken as independent predictors regarding bovine fasciolosis prevention and control (Table 5). The final model was an adequate fit to the data based on the results of the Hosmer and Lemeshow test (*χ*^2^ = 2.686, df = 4, *P* = 0.612).

**Table 3:** Univariate associations between a positive epidemiological knowledge score (score > 0 yes versus no) and potential covariates of smallholder cattle farmers in communal areas of North West Province South Africa from June to Oct 2019

Variable	Level	Parameter estimate($\widehat{{{\beta}}}$)	Odds ratio (95% CI)	*P* value
Location	Village M	0.304	1.36 (0.59, 3.10)	0.471
	Village T	0.043	1.04 (0.41, 2.66)	0.929
	Village G	Referent		
Individual	Owner	0.790	2.20 (0.78, 6.20)	0.135
	Attendant	Referent		
Sex	Male	0.225	1.25 (0.46, 3.40)	0.658
	Female	Referent		
Age	< 60 years	− 0.177	0.84 (0.33, 2.12)	0.709
	60–69 years	0.129	1.14 (0.47, 2.76)	0.776
	≥ 70 years	Referent		
Marital status	Married	− 0.760	0.47 (0.16, 1.36)	0.163
	Widow	− 0.624	0.54 (0.13, 2.25)	0.394
	Single or divorced	Referent		
Education	No formal education	Referent		
	Primary	− 0.090	0.91 (0.32, 2.59)	0.866
	Secondary or tertiary	− 0.100	0.91 (0.32, 2.55)	0.850
Language	Sepedi	0.258	1.29 (0.61, 2.77)	0.506
	Xhosa, Zulu, Afrikaans, or English	0.542	1.72 (0.59, 5.00)	0.320
	Other language	Referent		
Experience	< 10 years	− 0.204	0.82 (0.31, 2.18)	0.685
	10–20 years	− 0.701	0.50 (0.23, 1.10)	0.083
	> 20 years	Referent		
Cattle breed	Brahman	0.105	1.11 (0.49, 2.51)	0.800
	Nguni, Bonsmara, or non-descript	− 0.148	0.86 (0.34, 2.20)	0.757
	Multiple breeds or other	Referent		
Management	Backyard	− 0.036	0.96 (0.32, 2.89)	0.948
	Extensive	0.086	1.09 (0.41, 2.93)	0.865
	Semi-intensive	Referent		
Feed source	Grazing only	− 0.132	0.88 (0.42, 1.84)	0.728
	Mixed or concentrate feeding	Referent		

*CI*, confidence interval

**Table 4:** Univariate associations between a positive practice score (score > 0 yes versus no) and potential covariates of smallholder cattle farmers in communal areas of North West Province South Africa from June to Oct 2019

Variable	Level	Parameter estimate ($\widehat{{{\beta}}}$)	Odds ratio (95% CI)	*P* value
Location	Village M	0.338	1.40 (0.65, 3.04)	0.391
	Village T	0.908	2.48 (1.06, 5.80)	0.036
	Village G	Referent		
Individual	Owner	− 0.256	0.77 (0.34, 1.74)	0.535
	Attendant	Referent		
Sex	Male	− 0.936	0.39 (0.16, 0.96)	0.041
	Female	Referent		
Age	< 60 years	0.318	1.38 (0.59, 3.18)	0.457
	60–69 years	0.091	1.10 (0.48, 2.51)	0.830
	≥ 70 years	Referent		
Marital status	Married	− 0.120	0.89 (0.31, 2.54)	0.824
	Widow	0.608	1.84 (0.46, 7.31)	0.388
	Single or divorced	Referent		
Education	No formal education	Referent		
	Primary	− 0.112	0.89 (0.34, 2.39)	0.824
	Secondary or tertiary	0.472	1.60 (0.61, 4.21)	0.338
Language	Sepedi	− 0.694	0.50 (0.25, 1.01)	0.053
	Xhosa, Zulu, Afrikaans, or English	− 0.345	0.71 (0.26, 1.96)	0.506
	Other language	Referent		
Experience	< 10 years	− 0.044	0.96 (0.36, 2.52)	0.929
	10–20 years	0.544	1.72 (0.83, 3.59)	0.147
	> 20 years	Referent		
Cattle breed	Brahman	0.170	1.19 (0.56, 2.53)	0.660
	Nguni, Bonsmara, or non-descript	0.652	1.92 (0.83, 4.44)	0.127
	Multiple breeds or other	Referent		
Management	Backyard	− 1.712	0.18 (0.06, 0.54)	0.002
	Extensive	− 1.689	0.19 (0.07, 0.51)	0.001
	Semi-intensive	Referent		
Equipment	Has some equipment	0.447	1.56 (0.76, 3.21)	0.224
	No equipment for management	Referent		
Feed source	Grazing only	− 0.965	0.38 (0.19, 0.77)	0.007
	Mixed or concentrate feeding	Referent		
Water source	River	− 0.591	0.55 (0.29, 1.06)	0.076
	Other source	Referent		

*CI*, confidence interval

**Table 5:** Multivariable associations between a positive practice score (score > 0 yes versus no) and potential covariates of smallholder cattle farmers in communal areas of North West Province South Africa from June to Oct 2019

Variable	Level	Parameter estimate ($\widehat{{{\beta}}}$)	Odds ratio (95% CI)	*P* value
Education	Secondary or tertiary	0.724	2.06 (1.01, 4.20)	0.046
	Less education	Referent		
Cattle breed	Nguni, Bonsmara, or non-descript	1.029	2.80 (1.16, 6.77)	0.022
	Other breeds	Referent		
Management	Backyard	− 2.174	0.11 (0.03, 0.38)	< 0.001
	Extensive	− 1.803	0.17 (0.06, 0.47)	< 0.001
	Semi-intensive	Referent		

*CI*, confidence interval

## Discussion

The data on the knowledge of smallholder cattle farmers with regard to bovine fasciolosis in South Africa are scant. The current study sought to understand smallholder cattle farmer’s level of knowledge, attitudes, and practices on the epidemiology of bovine fasciolosis, which is an important task before embarking on any intervention strategies to control this parasitic disease in their herds. Farmers’ demographic structure was similar to the findings of Katikati and Fourie (2019) in a study on improving management practices of emerging cattle farmers in selected areas of the Eastern Cape Province of South Africa. The finding that most respondents were older farm owners with more than 10 years of cattle-rearing experience might be due to rural–urban migration where the elderly are left to farm and the more active youth seek employment and educational opportunities in urban areas (Mlambo 2018; Njwambe et al. 2019; Tada et al. 2012). This agrees with Oladele et al. (2013) who also reported a similar trend in the predominance of older more experienced farmers in selected villages in the same province. With the increase in unemployment levels in South Africa, more career guidance should be given to rural youth to encourage participation in cattle production.

More males were observed to be involved in cattle farming than females in the current study, most likely due to cattle operations often requiring physically demanding work. This is consistent with the findings of Chah et al. (2013) and Idamokoro et al. (2019) who also reported more males than females participating in livestock farming in rural villages of South Africa. The low level of education attained by farmers observed in the present study may likely be because of limited opportunities for higher level education in the rural settings where most smallholder farmers operate. This finding is similar to Yawa et al. (2020)’s report of low levels of education among cattle farmers in communal areas in the Eastern Cape Province of South Africa.

Cattle herd characteristics observed in the present study were typical of a communal livestock setting (Mapiye et al. 2009). The small herd sizes and abundant crossbred cattle might indicate low socio-economic status and lack of basic infrastructure necessary for the survival of improved exotic breeds. This agrees with the reports of Scholtz et al. (2008) who also reported an abundance of crossbred or non-descript cattle in South Africa. The small herd sizes in this study agree with the findings of Mapiye et al. (2009) who reported low cattle numbers per household in a communal farming setting of South Africa. The lack of basic farm equipment in virtually all herds and the reported average to poor body condition score of cattle observed likely indicate the poor socio-economic status of the sampled smallholder farmers. These findings agree with the reports of Schwalbach et al. (2001) who reported similar lack of farm infrastructure due to farmers’ low socioeconomic status in the North West Province of South Africa.

There were no significant associations between independent predictors evaluated and the epidemiological knowledge score concerning bovine fasciolosis among the smallholder cattle farmers studied. This could be due to many similarities between the farmers in the study areas; socio-demographic structure, herd structure, and climatic conditions were similar in all the villages. This finding agrees with that of Deka et al. (2020) who reported no significant association between farmers’ location and their knowledge score on zoonotic diseases in India. These findings also corroborate observations of Çakmur et al. (2015) who also reported no significant difference among farmers’ knowledge of zoonotic diseases and most independent predictors in Kars, Turkey.

The lack of significant predictors suggests that the level of knowledge in sampled communities is relatively unpredictable and that they possessed random level of knowledge. This could also indicate a general lack of training, dearth of training materials, and absence of knowledgeable people in the study area. The general lack of knowledge concerning bovine fasciolosis among smallholder cattle farmers observed in this present study might also be due to the asymptomatic nature of the disease in cattle or inadequate veterinary extension services in the area. Most farmers possessed low educational qualifications, which might limit their exposure and awareness about bovine fasciolosis. In a similar manner, several studies have reported poor knowledge among farmers in terms of transmission, prevention, and control of zoonoses (Cakmur et al. 2015; Hundal et al. 2016; Singh et al. 2019). This result is consistent with previous recommendations that the most effective intervention strategies to increase cattle farmer’s knowledge of animal diseases are continuous ‘on-the-job’ and ‘informal training’ (Nampanya et al. 2012). Munyeme et al. (2010) have attributed this low/random level of knowledge to remoteness, low training status on rearing and handling of animals, lack of health facilities, poor extension services, and low literacy rate among cattle farmers.

The association between farmers’ positive practice scores and independent predictors is similar to what Çakmur et al. (2015) reported in their work on the assessment of farmers’ practices concerning zoonotic diseases in Kars, Turkey. A previous study also reported a positive influence of farmers’ educational status, income levels, and size of enterprise on their knowledge, attitude, and practices toward zoonotic diseases (Özlü et al. 2020). Furthermore, Moutos et al. (2022) reported that ruminant farmers’ level of education and extent of veterinary supervision were the only independent predictors for their evaluated practice scores in the assessment of knowledge related to zoonotic diseases in Elassona Municipality, Greece. Positive associations were also reported between farmers’ age and educational status and increased practice scores related to antibiotics use and resistance among animal farm owners/workers in Amhara region, north western Ethiopia (Geta and Kibret 2021).

Farmers’ educational level, system of management, and cattle breeds farmed were the predictors that were retained in the final multivariable model in the present study. There was a higher likelihood that farmers that attained higher education level had more positive practices that helped in the prevention and control of bovine fasciolosis. This finding is similar to the observation of Sadiq et al. (2021), who reported that ruminant farmers with higher educational qualifications have better knowledge to implement practices against zoonotic diseases in Selangor, Malaysia. Smallholder farmers that owned Nguni, Bonsmara, or non-descript breeds of cattle also had a higher likelihood of improved practices about the prevention and control of bovine fasciolosis compared to farmers that reared other breeds. This finding might be associated with few numbers of Nguni, Bonsmara, and non-descriptive breeds in the study population. It could also be due to long years of rearing experience by the smallholder farmers owning these breeds, as Nguni, Bonsmara and other non-descriptive breeds have been reported to possess higher adaptability, higher resilient ability to ticks, tick-borne diseases, and nematodes feed (Ndlovu 2007; Muchenje et al. 2008). Nguni breeds also have improved feed efficiency and better ability to select improved quality diets from coarse forages on rangelands (Collins-Lusweti 2000). However, the years of experience were not a significant predictor of improved practices, and thus, the link between cattle breed and improved practices might be more complex and possibly a proxy for unmeasured variables in the study.

Farmers that employed a semi-intensive management system had a higher likelihood of implementing positive bovine fasciolosis preventive strategies compared to those farmers engaging in backyard or extensive systems of management. The farmers engaging in semi-intensive management system were possibly more likely to seek education and intervention from veterinary personnel. All preventable measures such as avoidance of water logged pasture, avoidance of early morning grazing, pasture management, rotational grazing, and periodical prophylactic treatment and routine deworming with anthelminthic might have been instituted because of a veterinary herd health program. Moutos et al. (2022) similarly reported on the importance of farmers’ education for the prevention of zoonotic diseases. Also, veterinary supervision, which might be more likely with semi-intensive systems, has been linked to improved practices for the prevention of zoonotic diseases. Gaps in knowledge and high-risk practices concerning bovine brucellosis have been associated with the absence of veterinary supervision in Portugal (Díez and Coelho 2013). It is thus important for smallholder farmers to be trained on the epidemiology of bovine fasciolosis to improve their knowledge and practices and thus reduce the negative impact of the disease on their herds. The significantly higher likelihood of a positive practice score regarding bovine fasciolosis in village T compared to village G may be due to the nearness of village T to a major city compared to village G. It may also be due to previous training or enriched extension services by the veterinary extension officer as a result of nearness. Possession of a lower likelihood of positive score by the male farmers compared to female farmers as observed in this study may be due to previous training that might have been received by the female farmers or relatively higher commitment of female farmers compared to male ones.

The present study’s findings should be interpreted in conjunction with several limitations because bias in questionnaire studies is inevitable. This is a fundamental issue in public health research and categorized in three ways: challenges associated with question design, whole questionnaire design, and administration of the questionnaire (Choi and Pak 2005). In the present study, bias was minimized by carefully designing each question and pre-testing conducted using farmers in a different location. Correct statements concerning bovine fasciolosis epidemiology and improved practices were mixed with some false statements to objectively assess farmers’ knowledge. Furthermore, bias such as response bias due to self-reporting was beyond the authors’ control, especially when the participant wanted to satisfy the researchers by participating in the survey (Rosenman et al. 2011). The findings of this survey might therefore suffer for some social desirability bias. The sample size, non-random selection of participants, and data collection via structured questions might not adequately represent the study population. Language also appeared to be a limitation as interpreters were required, which might not have translated the questions correctly. More so, some farmers were not patient enough to listen attentively before offering their responses. Notwithstanding the potential limitations, important data and findings have been collected and reported in the current study.

## Conclusions

The present study identified that smallholder cattle farmers, especially less educated farmers and extensive producers, in the North West Province had poor likelihood of executing satisfactory practices on the prevention and control of bovine fasciolosis. Training and awareness sessions for smallholder farmers on these aspects are therefore recommended.

## Data Availability

The datasets generated during the current study are available from the corresponding author on request.
